# High-intensity infrasound effects on glucose metabolism in rats

**DOI:** 10.1038/s41598-021-96796-5

**Published:** 2021-08-26

**Authors:** Gonçalo Martins Pereira, Madalena Santos, Sofia S. Pereira, Gonçalo Borrecho, Francisco Tortosa, José Brito, Diamantino Freitas, António Oliveira de Carvalho, Artur Águas, Maria João Oliveira, Pedro Oliveira

**Affiliations:** 1 Center for Interdisciplinary Research Egas Moniz (CiiEM), Quinta da Granja, Monte da Caparica, 2829-511 Caparica, Portugal; 2 Department of Anatomy and UMIB–ITR (Unit for Multidisciplinary Research in Biomedicine - Laboratory for Integrative and Translational Research in Population Health), ICBAS (Instituto de Ciências Biomédicas Abel Salazar), Universidade Do Porto, Porto, Portugal; 3 Laboratory of Acoustics, Faculty of Engineering (FEUP), University of Porto, Porto, Portugal

**Keywords:** Animal disease models, Endocrine system and metabolic diseases, Pathogenesis

## Abstract

Recent focus has been given on the effects of high-intensity infrasound (HII) exposure, and whether it induces changes in pancreatic morphology and glucose metabolism is still unknown. As such, we have studied the impact of HII exposure on glucose tolerance, insulin sensitivity, pancreatic islet morphology, muscle GLUT4 and plasma insulin and corticosterone levels. Normal and glucose intolerant wild-type Wistar rats were randomly divided in two groups: one group not exposed to HII and the other continuously exposed to HII. Animals were sacrificed at three timepoints of exposure (1, 6 or 12 weeks). An intraperitoneal glucose tolerance test was performed, blood samples were collected and the pancreas and the quadriceps femoris muscle were excised. Circulating insulin and corticosterone levels were determined and pancreatic and muscular tissue were routinely processed for histochemistry and immunohistochemistry with an anti-GLUT4 antibody. Animals exposed to HII had higher corticosterone levels than animals not exposed. No differences were found on insulin concerning HII exposure or glucose intolerance. Glucose intolerant animals had pancreatic islet fibrosis and no differences were found in GLUT4 ratio concerning HII exposure. In conclusion, we found that continuous exposure to HII increases stress hormone levels without inducing glucose intolerance in rats.

## Introduction

Noise pollution is a ubiquitous hazard, present in environmental and occupational settings, known to cause adverse effects on human health^1^. Among the sound spectrum, there has been a great interest on the health effects of high-intensity infrasound (frequency < 20 Hz and a sound pressure level > 90 dB) exposure in the general population^2^. Due to their long wavelength, infrasonic frequencies are hardly attenuated through dissipation and can induce body vibrations and resonance in body cavities, thus affecting internal systems and organs^3,4^.

In humans, exposure to acoustical environments rich in high-intensity infrasound (such as the ones present in industrial settings) has been shown to cause extra-auditory effects such as annoyance, sleep disturbance, psychological stress, and cardiovascular disease, including ischemic cardiomyopathy, heart failure, hypertension, arrythmia and stroke^5^. Experimental studies in rats report an increase of collagen fibres without inflammatory signs in blood vessel walls, trachea, lungs, and serous membranes^6^. Other findings include impaired hippocampus-dependent learning and memory^7^, loss of tracheal and bronchial ciliated cells^8,9^, decrease in pleural microvilli^10^, myocardial dysfunction and decrease in cardiac connexins^11,12^, disruption of salivary glands acinar structure with quantitative and qualitative alterations in saliva^13^ and cytological changes in adrenal glands suggestive of increased steroidogenic activity^14^.

Systematic reviews and meta-analysis have identified an increased risk of type 2 diabetes mellitus associated with noise exposure in a time-dependent manner, with a stronger association for long-term exposure^15–18^. However, considering the different frequencies in the sound spectrum, whether high-intensity infrasound exposure induces changes in glucose metabolism is still unknown. For this reason, we have studied the morphophysiological impact of high-intensity infrasound exposure on glucose metabolism of normal and glucose intolerant rats. We measured glucose tolerance, insulin sensitivity, pancreatic islet morphology and fibrosis, skeletal muscle GLUT4 ratio and plasma insulin levels. We have also studied the impact of this exposure on the rat hypothalamic–pituitary–adrenal endocrine axis, a main element in stress response, by measuring plasma corticosterone levels.

## Results

### Effects of high-intensity infrasound on glucose tolerance, insulin sensitivity and insulin response during IPGTT in rats

**Figure 1:**
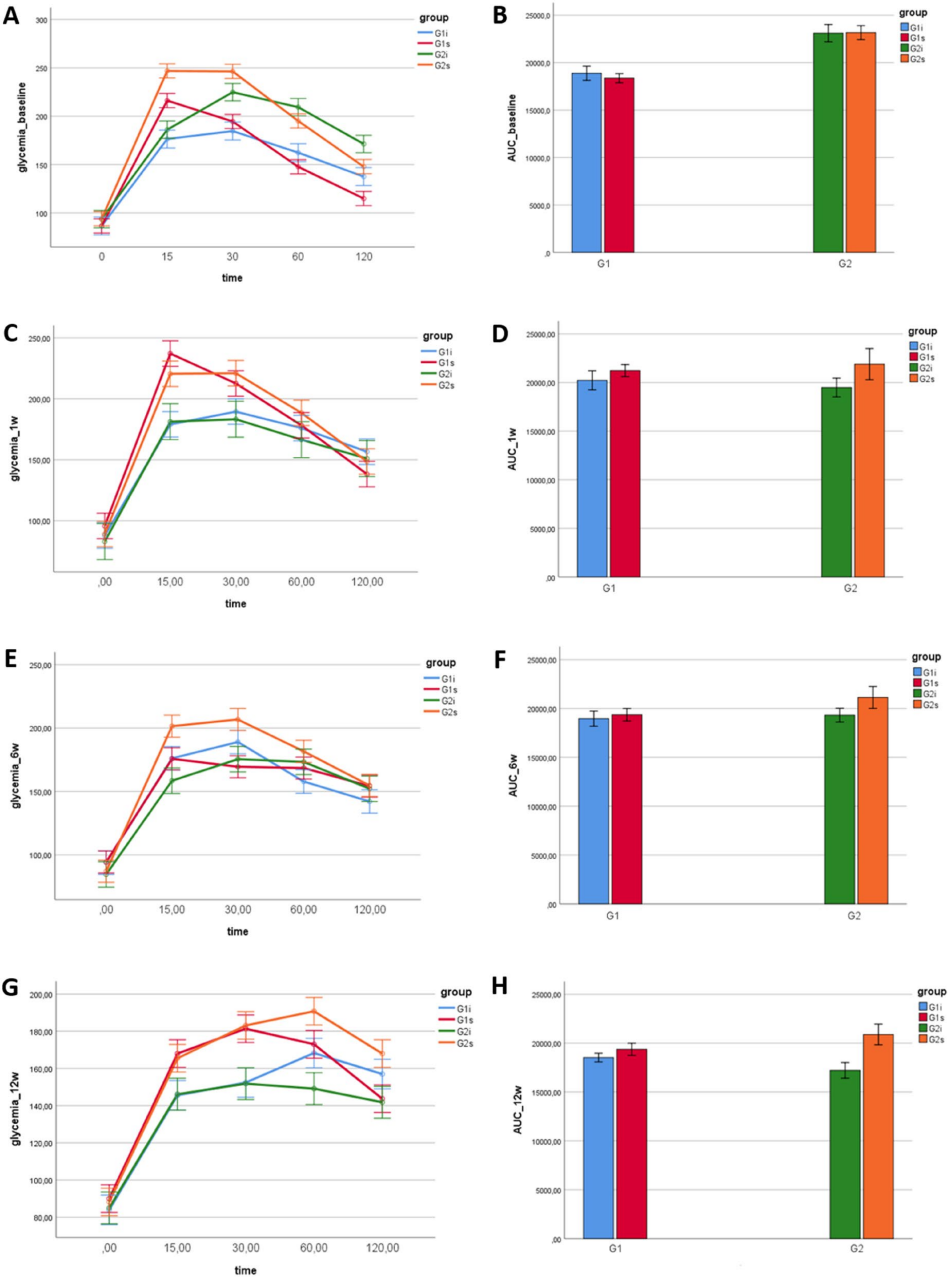
Intraperitoneal glucose tolerance test curve and respective area under the curve (AUC). Means ± SE for each timepoint of the intraperitoneal glucose tolerance test curve (**A**, **C**, **E**, **G**) and the respective glucose AUC (**B**, **D**, **F**, **H**) of each experimental group of normal (G1) and glucose intolerant (G2) animals, either kept in silence (s) or exposed to high-intensity infrasound (i).

Concerning glucose intolerance before high-intensity infrasound exposure, we found that G2, glucose intolerant, animals presented higher glycemia than G1, normal, animals (*p* < 0.001). Baseline glycemia of the intraperitoneal glucose tolerance test, at the 2 h timepoint, for G1 group had a mean value of 124 mg/dL ± 18.4 mg/dL and for G2 group had a mean value of 158 mg/dL ± 30.6 mg/dL (Table 1). Glucose AUC respective to the baseline was higher in G2, when compared to G1 (*p* < 0.001), thus G2 animals were considered glucose intolerant (Fig. 1B).

**Table 1:** Distribution of animals per experimental groups.

G1 (n = 28)			G2 (n = 28)		
124 ± 18.4 mg/dL			158 ± 30.6 mg/dL		
G1s (n = 14)	1 wk (n = 4)	138 ± 13.8 mg/dL	G2s (n = 14)	1 wk (n = 4)	149 ± 45.5 mg/dL
	6 wks (n = 5)	154 ± 20.5 mg/dL		6 wks (n = 5)	155 ± 17.4 mg/dL
	12 wks (n = 5)	144 ± 21.3 mg/dL		12 wks (n = 5)	168 ± 25 mg/dL
G1i (n = 14)	1 wk (n = 4)	157 ± 27.8 mg/dL	G2i (n = 14)	1 wk (n = 4)	151 ± 17.6 mg/dL
	6 wks (n = 5)	142 ± 20.8 mg/dL		6 wks (n = 5)	152 ± 22.3 mg/dL
	12 wks (n = 5)	142 ± 14.2 mg/dL		12 wks (n = 5)	157 ± 11.5 mg/dL

Mean values and standard deviation for glycemia at the intraperitoneal glucose tolerance test 2 h timepoint, expressed as milligrams per deciliter (mg/dL), for each experimental group of normal (G1) and glucose intolerant (G2) animals, either kept in silence (s) or exposed to high-intensity infrasound (i).

No differences were found in final glucose AUC between G1, normal, and G2, glucose intolerant rats (*p* = 0.907), after controlling for baseline glucose AUC. However, animals not exposed to high-intensity infrasound presented higher glucose AUC than exposed animals (*p* = 0.030), regardless of their glucose tolerance status and after removing the effects of baseline insulinemia. Animals exposed to 1 week of high-intensity infrasound presented higher glucose AUC when compared to animals exposed to 12 weeks of high-intensity infrasound (*p* = 0.047) (Fig. 1).

**Figure 2:**
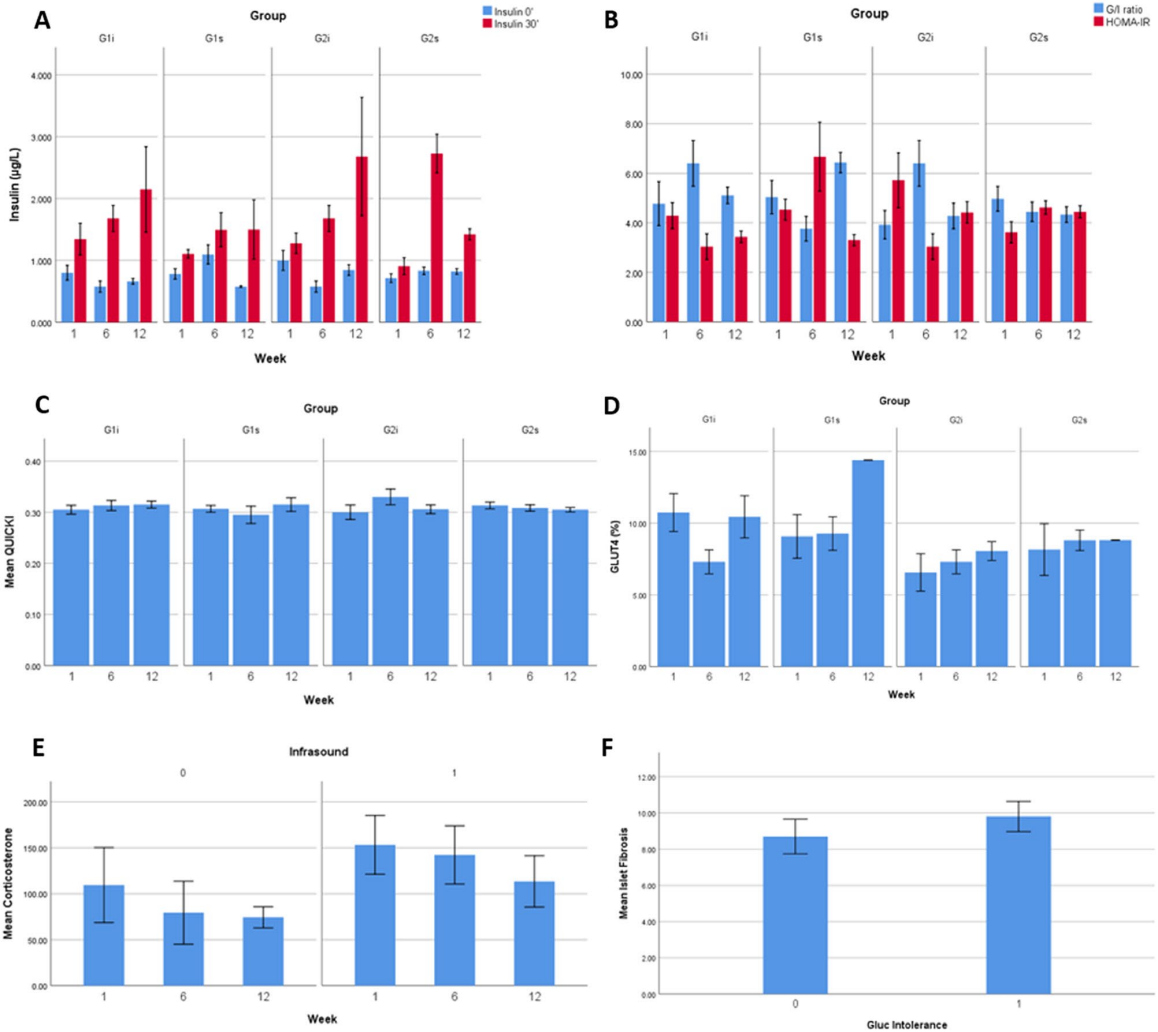
Plasma insulin and corticosterone levels, glucose/insulin ratio, HOMA-IR, QUICKI, muscle GLUT4 ratio and islet fibrosis ratio. Means ± SE of plasma insulin levels (**A**), glucose/insulin (G/I) ratio (**B**), HOMA-IR (**B**), QUICKI (**C**), muscle GLUT4 ratio (**D**), plasma corticosterone levels (**E**) and pancreatic islet fibrosis ratio (**F**), in normal (G1) and glucose intolerant (G2) animals, either kept in silence (s) or exposed to high-intensity infrasound (i), throughout the established experimental timepoints.

Regarding plasma insulin levels, no differences were found on plasma insulin levels concerning infrasound exposure (*p* = 0.531) or glucose intolerance (*p* = 0.518). After controlling for infrasound exposure duration, no differences were found between fasting insulin levels and insulin levels 30 min after glucose administration (*p* = 0.124) (Fig. 2A).

Regarding glucose/insulin ratio, no differences were found concerning infrasound exposure (*p* = 0.596) or glucose intolerance (*p* = 0.883) (Fig. 2B). Also, no differences were found on HOMA-IR regarding infrasound exposure (*p* = 0.318) or glucose intolerance (*p* = 0.402) (Fig. 2B). Similarly, no differences were found on QUICKI regarding infrasound exposure (*p* = 0.163) or glucose intolerance (*p* = 0.464) (Fig. 2C).

### Analysis of plasma corticosterone levels in rats

Considering plasma corticosterone levels, no differences were found between glucose tolerant and glucose intolerant animals (*p* = 0.674). However, animals exposed to high-intensity infrasound presented higher plasma corticosterone levels than animals not exposed (*p* = 0.043) (Fig. 2E).

### Effects of high-intensity infrasound on insulin signaling in rat muscle

Regarding the quantity of GLUT4 transporter in skeletal muscle, G2 animals presented lower values than G1 animals (*p* = 0.002). This finding supports the fact that G2 animals were indeed glucose intolerant, as confirmed by the baseline and subsequent intraperitoneal glucose tolerance tests. No differences were found in muscle GLUT4 ratio concerning high-intensity infrasound exposure (*p* = 0.506) or concerning duration of high-intensity infrasound exposure (*p* = 0.230) (Fig. 2D).

### Effects of high-intensity infrasound on rat pancreatic and muscle tissue morphology

**Figure 3:**
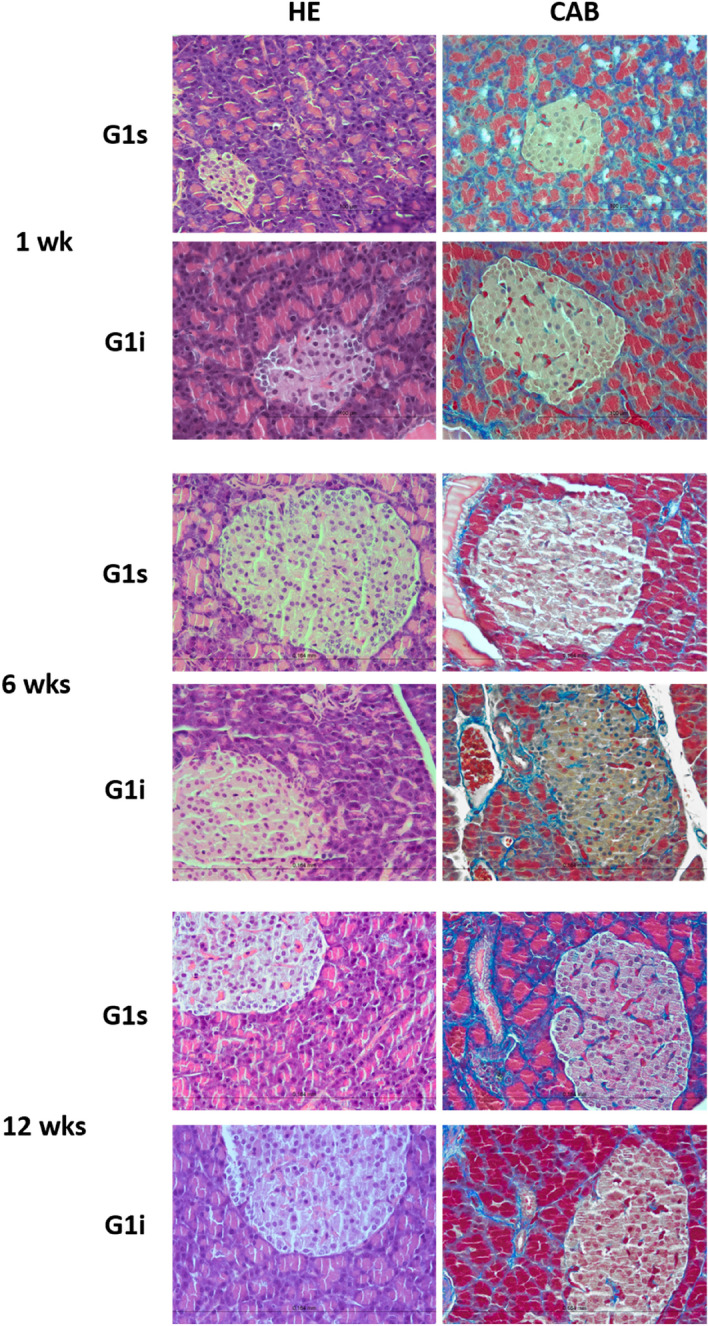
Pancreatic morphology of normal rats. Representative images of pancreatic sections (40 × objective), stained with hematoxylin–eosin (HE) and chromotrope aniline blue (CAB), of normal (G1) animals, either kept in silence (s) or exposed to high-intensity infrasound (i), throughout the established timepoints.

The pancreatic tissue of normal animals, G1 group, both kept in silence and exposed to high-intensity infrasound, presented a regular micro-anatomy without cellular alterations throughout the established timepoints. Slight alterations and a small increase in the quantity of collagen fibers, both in the pancreatic islets and the exocrine parenchyma, was observed at the 6-week and 12-week timepoint in animals kept in silence (G1s) and animals exposed to high-intensity infrasound (G1i). This fibrosis on the endocrine and exocrine parenchyma was considered medium degree and was more pronounced in the glucose intolerant group (Fig. 3).

**Figure 4:**
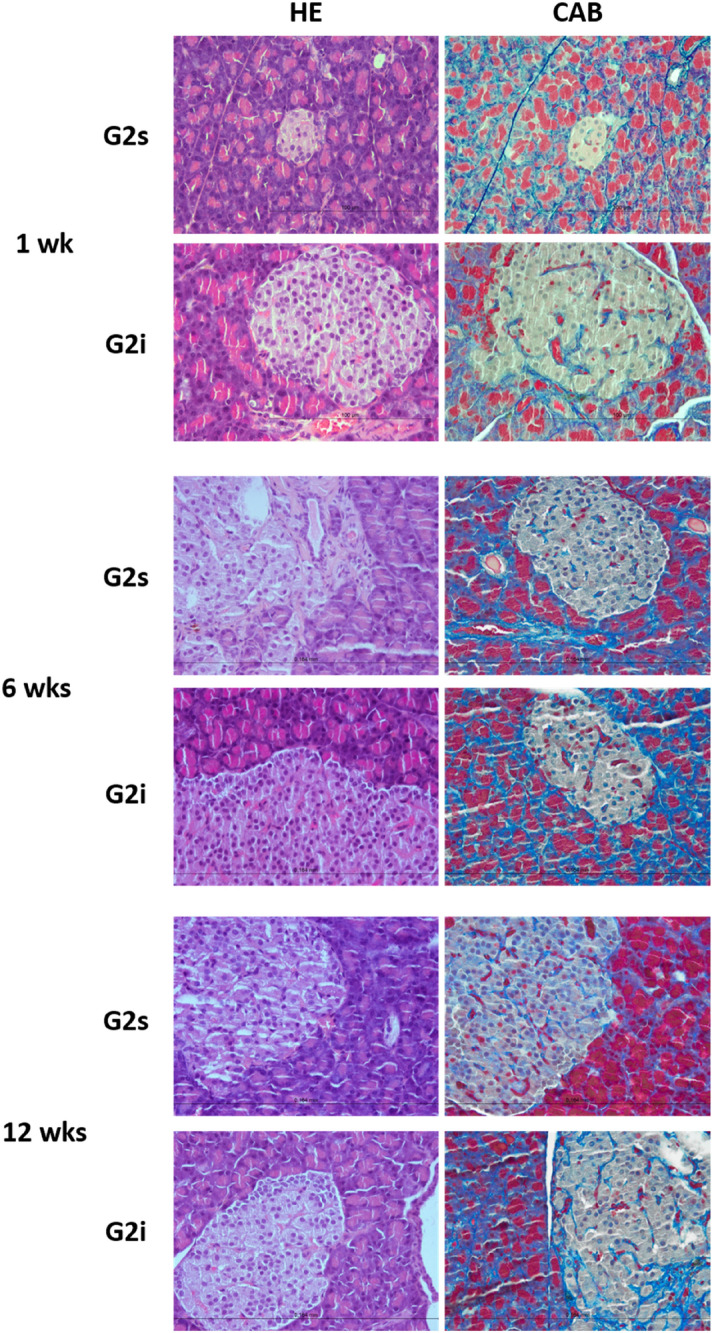
Pancreatic morphology of glucose intolerant rats. Representative images of pancreatic sections (40 × objective), stained with hematoxylin–eosin (HE) and chromotrope aniline blue (CAB), of glucose intolerant (G2) animals, either kept in silence (s) or exposed to high-intensity infrasound (i), throughout the established timepoints.

In glucose intolerant animals, G2 group, both kept in silence and exposed to high-intensity infrasound, the pancreas presented regular micro-anatomy, scarce collagen fibers and slight capillary congestion at the 1-week timepoint. A moderate increase in the quantity of collagen fibers in the pancreatic islets was observed in glucose intolerant animals not exposed to high-intensity infrasound (G2s) at the 6- and 12-week timepoint with a severe increase in the quantity of collagen fibers in the exocrine parenchyma at the same timepoints. Glucose intolerant animals exposed to high-intensity infrasound (G2i) had a more severe fibrosis, mainly in the periductal islets, than their counterparts kept in silence throughout the established timepoints. No cellular alterations were observed in glucose intolerant animals kept in silence and exposed to high-intensity infrasound at any timepoint (Fig. 4).

Histochemical analysis of pancreatic islet fibrosis show that animals exposed to high-intensity infrasound did not present different levels of pancreatic islet fibrosis when compared to animals not exposed (*p* = 0.651). However, higher pancreatic islet fibrosis was found in glucose intolerant animals, when compared to glucose tolerant animals (*p* = 0.007), after removing the effects of exposure duration (Fig. 2F).

**Figure 5:**
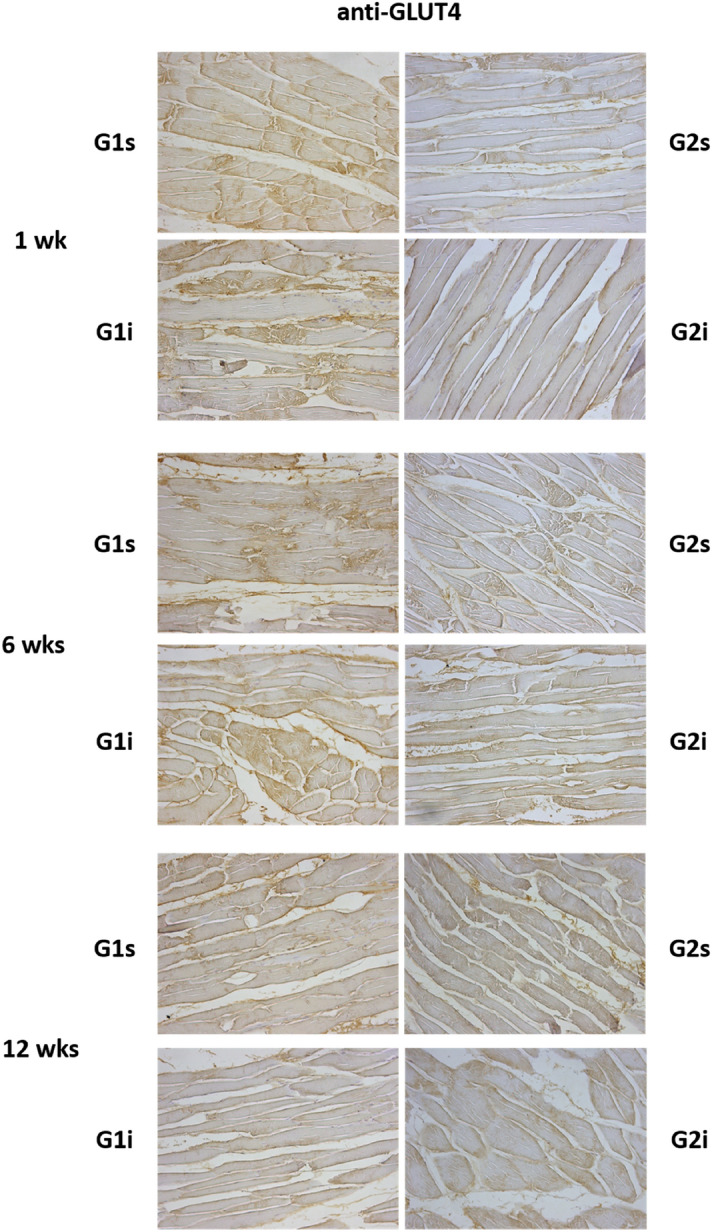
Muscle morphology and anti-GLUT4 immunohistochemical analysis in rats. Representative images of quadriceps femoris muscle sections (40 × objective), with immunohistochemical staining using anti-GLUT4 specific antibody, of normal (G1) and glucose intolerant (G2) animals, either kept in silence (s) or exposed to high-intensity infrasound (i), throughout the established timepoints. GLUT4 is stained brown.

Regarding skeletal muscle morphology, no differences were found between groups in different timepoints, and all animals had a regular micro-anatomy (Fig. 5). Muscle fibers were organized in bundles and the nuclei were located peripherally. The sarcoplasm appeared uniform and no cellular alterations, inflammatory cell infiltration, fibrosis or necrosis were identified.

## Discussion

Our morphophysiological study aimed to investigate whether continuous exposure to infrasonic noise was able to induce alterations in glucose metabolism and pancreatic morphology in rats. Results showed that exposure to this environmental and occupational hazard increases plasma corticosterone levels, without inducing alterations in glucose tolerance, insulin levels or peripheral insulin sensitivity. Also, no morphological changes were observed in pancreatic and skeletal muscle tissue due to exposure to high-intensity infrasound.

The mechanisms by which infrasonic noise affects biological systems are still unclear. Currently there are two main proposed models for the systemic damage induced by high-intensity infrasound exposure. One model relates to the annoyance induced by exposure to this aggressor and the sustained reactive neuroendocrine stress response. The other relates to the mechanic effects of body vibrations and resonance of internal organs induced by high-intensity infrasound that may lead to physical disruption of tissues^6,19^. Whether both models act independently or simultaneously is still unknown.

Considering the first model, high-intensity infrasound acts as a physical and psychological stressor, triggering the neuroendocrine sympatho–adrenomedullary and hypothalamic–pituitary–adrenal axes that increase corticosterone and catecholamines and, thus, promote hyperglycemia^20^. Chronic exposure to corticosterone can lead to chronic hyperglycemia and, as such, promote hyperinsulinism and insulin resistance^21,22^. Previous studies in rats have shown that exposure to industrial noise, rich in high-intensity infrasound, decreases the volume of the adrenal zona fasciculata together with depletion of adrenal lipid droplets, both suggestive of increased stimulation of adrenal steroidogenesis of glucocorticoids^14^. In our study we have found that high-intensity infrasound increases plasma corticosterone levels, the main stress hormone in rats^23^. Although the intraperitoneal route used to assess glucose tolerance increases corticosterone levels in rats^24^, since all rats were submitted to the same protocol, the stress induced by the IPGTT was similar in all experimental groups and, therefore, unlikely to influence our data. The fact that no hyperinsulinism and insulin resistance were observed as a result from exposure to high-intensity infrasound and from increased plasma corticosterone levels could point to a delayed response to this stressor and we argue that a longer period of exposure would be needed to observe changes in insulin production, insulin resistance and glucose tolerance^25^, as seen in type 2 diabetes mellitus pathophysiology^26^.

Animal exposure studies focusing on audible noise with higher frequencies found an increase in insulin resistance, fasting hyperglycemia, dyslipidemia, and alterations in insulin signaling in the skeletal muscle^25,27–29^. Other studies show that exposure to high-frequency audible noise has no influence in glucose tolerance^25,28^. Our animals exposed to high-intensity infrasound had lower glucose AUC, and thus higher glucose tolerance, than their counterparts kept in silence. We interpret this finding as a reactive response, by the pancreas and/or the peripheral insulin receptors and insulin-regulated transporters, that occurs in the presence of this aggressor. In fact, compensatory increases in insulin production and pancreatic islets beta cell mass^30^ have been reported as a response to several injury models, whether by enhanced function or increase in the number of beta cells by self-replication or conversion of other pancreatic islet cell types to beta cells^31^. Nevertheless, we have not found cellular alterations in the endocrine pancreas or differences on plasma insulin due to high-intensity infrasound exposure.

Concerning a possible reactive response in peripheral insulin-regulated glucose transporters, we have not found significant differences in muscle GLUT4 ratio between animals exposed to high-intensity infrasound and animals kept in silence. This finding suggests that alterations in other peripheral insulin receptors or insulin-regulated glucose transporters could be at play, namely on adipose and hepatic tissue^32^. The liver plays an important part in glucose homeostasis through insulin clearance, maintaining a homeostatic level of insulin^33^. However, the chronic hyperinsulinism that accompanies increased beta cell mass also leads to hepatic steatosis^33^ and high-intensity infrasound does not appear to alter the hepatic lipid content of both normal and glucose intolerant animals^34^.

Considering the direct effects of high-intensity infrasound in tissues, other exposure animal studies showed that continuous exposure results in proliferation of collagen fibers and tissue fibrosis^12,13^. Several authors suggest that the body vibrations and resonance produced by high-intensity infrasound represents a mechanical stimulus that activates intracellular signaling pathways resulting in extracellular matrix remodeling and fibrosis^6,35^. This mechanism, called mechanotransduction, may function as a mechanical stabilizer of the organ^6,35^. Oliveira et al.^13^ documented perivasculo-ductal fibrosis in the rat parotid gland, as well as disruption of acinar structure, generalized vacuolization and signs of necrosis that were associated with quantitative and qualitative alterations in saliva production. Due to certain anatomical and functional similarities between the parotid gland and the pancreas^36^, similar effects could also be expected due to high-intensity infrasound exposure. However, we have not observed an increase in collagen fiber quantity in pancreatic islets with exposure to this stressor. The rat pancreas presents several segments with different macroscopic appearance, from a relatively compact splenic segment to a duodenal segment dispersed within the mesentery^37^. We argue that the deep location of the rat pancreas segments in the abdominal cavity could protect this organ from body vibration due to high-intensity infrasound and thus from tissue damage^14^.

The fibrosis found in pancreatic islets of glucose intolerant animals could be either the result of glucose intolerance or the result of the glucose intolerance method used in this study. Glucose intolerance has an aggravating effect on pancreatic fibrosis caused by chronic inflammation from other pancreatic pathologies^38^. Regarding the used method, streptozotocin displays selective pancreatic beta cell toxicity and induces beta cell death^39^. This process induces tissue injury and local inflammatory response that activates pancreatic stellate cells with subsequent extracellular remodeling and deposition of collagen fibers^40,41^.

Future studies should appraise the ultrastructural effects of high-intensity infrasound exposure in the pancreatic and skeletal muscle, through transmission electron microscopy^42^. Although immunohistochemistry allows the quantification of muscle GLUT4, other quantitative methods such as western blot could be of use, due to their greater reproducibility and sensibility^43^. Besides the intraperitoneal route used to determine glucose tolerance, the use of the oral route would provide information regarding the incretin effect, and thus allow a better understanding of the pancreatic insulin release^44^. A longer period of high-intensity infrasound exposure would allow us to better observe changes in glucose metabolism and refine the understanding of the pathophysiology of infrasound-induced metabolic dysfunction and type 2 diabetes mellitus^25^. Since high-intensity infrasound exposure effects on glucose metabolism are still unknown, a positive control was difficult to establish for this acoustic element. Our results will help establishing positive controls in future studies. Despite the small number of animals per group, in each of the 12 experimental groups, the number of animals in each group was sufficient to perform the adequate statistical analysis for the chosen design.

In conclusion, continuous exposure to high-intensity infrasound increases corticosterone levels, without inducing glucose intolerance and alteration of plasma insulin or peripheral insulin sensitivity through GLUT4 transporter. This environmental stimulus can act as a cofactor in metabolic dysfunction and type 2 diabetes mellitus in a time-dependent manner. These results highlight the importance of further research concerning the metabolic effects of high-intensity infrasound, due to its ubiquitous diurnal and nocturnal presence in a common daily life and its possible action in type 2 diabetes mellitus pathophysiology, whose importance is yet to be determined.

## Methods

### Animals

Study design and sample size estimation were carried out as described previously^34^. In short, fifty-six wild-type male Wistar rats were acquired from Charles River Laboratories (Saint-Germain-sur-l’Arbresle, France), aged 11 weeks, and with an average weight of 376 g ± 18.3 g. Only male rats were included to avoid uncertain sex-dependent differences on the outcomes. Upon arrival and throughout the entire study, the animals were kept in standard cages, exposed to a light/dark cycle of 12 h and had free access to food and water. All animals passed the Preyer reflex test, a simple method to estimate auditory function^45^.

After a one-week acclimation period the animals were randomly assigned using a free access online software^46^ into two groups: G1 (no treatment, n = 28) and G2 (glucose intolerance, n = 28). Glucose intolerance was induced through a high-fat diet and the administration of low-dose streptozotocin (HFD/STZ rat model) because this model is considered to mimic the human disease^47^. G1 and G2 animals were then fed standard rat chow, to eliminate further differences between groups, and were randomly assigned into two subgroups each: G1s (no treatment, silence, n = 14), G1i (no treatment, infrasound, n = 14), G2s (glucose intolerance, silence, n = 14) and G2i (glucose intolerance, infrasound, n = 14). Animals from G1i and G2i were continuously exposed to high-intensity infrasound while G1s and G2s animals were kept in similar conditions but without high-intensity infrasound exposure. Intraperitoneal glucose tolerance tests were performed and animals euthanized in all experimental groups after 1, 6 or 12 weeks of high-intensity infrasound exposure. Animals were randomly distributed as shown in Table 1.

Experimental design and planning were performed with full compliance to the PREPARE guidelines^48^. Animal procedures were approved by the Portuguese National Authority for Animal Health and the local Animal Welfare Body (project nº 204/2017). All handling and care of the animals was performed humanely and alleviating animal suffering by authorized researchers (accredited with FELASA Category C) and was done in accordance with the EU Commission on Animal Protection for Experimental and Scientific Purposes (2010/63/EU) and with the Portuguese legislation for the same purpose (DL 113/2013). The study was carried out in compliance with the ARRIVE guidelines^49^.

### Glucose intolerance

Glucose intolerance was induced following the protocol by Furman^50^. Animals were fed a high-fat diet (D12492 diet, Research Diets Inc., USA) for 3 weeks. On a caloric basis, the high-fat diet consisted of 60% fats, 20% carbohydrates and 20% protein (5.21 kcal/g energy density) whereas the standard rat chow (D10001 diet, Research Diets Inc., USA) consisted of 12% fats, 67% carbohydrates and 21% protein (3.86 kcal/g energy density). After three weeks of high-fat diet, low-dose streptozotocin (STZ, Sigma-Aldrich, USA) 40 mg/kg was prepared in a sodium citrate buffer 50 mM, pH4.4, and was administered intraperitoneally after a fasting period of 6–8 h. Animals had unlimited access to water during fasting period.

Glucose intolerance was confirmed through an intraperitoneal glucose tolerance test following the protocol established by Ayala et al.^51^. Animals were considered glucose intolerant if they presented a plasma glucose > 140 mg/dL at the 2 h timepoint of the test^51^. The results of the intraperitoneal glucose tolerance test were expressed as the baseline area under the curve (AUC) for each animal.

### High-intensity infrasound exposure

High-intensity infrasound exposure was performed as previously published^52^. In short, pseudo-random waveform in the 2 to 20 Hz decade band was designed with Matlab based on a bandpass-filtered 30-s maximum length sequence segment. The total sound pressure level and the spectral characteristics of the resulting acoustic pressure waveform were monitored throughout the experiment and showed an average sound pressure level of 120 dB ± 3 dB in the 30-s time window. The exposure was continuous (24 h/day) to reflect the ubiquitous diurnal and nocturnal presence of high-intensity infrasound in a common daily life^53^.

### Intraperitoneal glucose tolerance test and plasma insulin

At the respective timepoint and before euthanasia, an intraperitoneal glucose tolerance test was performed, following the protocol established by Ayala et al.^51^. Animals were fasted overnight for 12 h and the fasting blood glucose level was determined through a sample collected from the caudal vein in a standard glucometer (Freestyle Precision, Abbott, USA). A 20% glucose solution was administered intraperitoneally (1 ml/Kg) and the blood glucose was measured at 15-, 30-, 60- and 120-min post-injection. The glycemia recorded during the test was used to evaluate glucose tolerance to the glucose challenge. The results of the intraperitoneal glucose tolerance test were expressed as both a time course of glycemia measurements and as the area under the curve (AUC) for each animal. The time course of glycemia measurements and the AUC was averaged for each experimental group (Fig. 1).

In addition to blood glucose measurements during the intraperitoneal glucose tolerance test, blood samples from the caudal vein were taken before and 30 min after glucose administration to assess plasma insulin levels. Following centrifugation, the resulting plasma was separated and kept frozen for later analysis. Plasma insulin levels were measured using commercially available ELISA kits (Rat Insulin ELISA kit 10–1250-10, Mercodia), according to the manufacturer’s instructions and guidelines. Insulin levels are expressed as micrograms per liter (µg/L). Fasting levels of glucose and insulin were used to calculate the glucose/insulin ratio, the homeostasis model assessment insulin resistance (HOMA-IR) and the quantitative insulin check index of insulin sensitivity (QUICKI), which are the most common indirect methods used to measure insulin sensitivity^54^.

### Tissue harvest

After the intraperitoneal glucose tolerance test, animals were euthanized by inhalation of carbon dioxide. This method is an acceptable method for rodent euthanasia and was performed considering the most recent recommendations^55^. Blood samples were collected and the pancreas and quadriceps femoris muscle were dissected, excised, and immersed in a 4% (vol./vol.) buffered paraformaldehyde solution.

### Plasma corticosterone

Blood collected at sacrifice was centrifugated and the resulting plasma was separated and kept frozen for later analysis. Plasma corticosterone levels were measured using commercially available ELISA kits (Corticosterone ELISA kit ab108821, Abcam), according to the manufacturer’s instructions and guidelines. Corticosterone levels are expressed as nanograms per milliliter (ng/mL) of plasma.

### Immunohistochemistry and morphological analysis

After routine processing for light microscopy, five micrometer paraffin-embedded slices of the pancreatic tissue sample were made and dyed according to the hematoxylin–eosin (HE) and chromotrope aniline blue (CAB) techniques. Histological images were acquired with a Leica DM500 light microscope and a Leica DFC290 HD camera (Leica Microsystems CMS GmbH, Wetzlar, Germany) using a 40 × objective. The morphological analysis was blinded to the groups and performed by an experienced endocrine pathologist, that examined the slides in random order and divided the observations as part of one of four groups: no alterations, slight, medium, and severe.

For the histochemical analysis of pancreatic islet fibrosis, three random images of equal area containing pancreatic islets were selected from each animal and analyzed using the ImageJ software^56^, as described previously^12^. In short, for each image the pancreatic islets were isolated, and their blue pixel content was measured relative to the total islet area using a color deconvolution method^57^. The non-staining sections in interstitial spaces were excluded from quantification. The ratio of fibrosis area to islet area was calculated and the mean ratio of the three images was obtained for each animal.

For the immunohistochemistry analysis, a standard immunohistochemical technique was followed as described previously^12^, using a rabbit polyclonal anti-GLUT4 antibody (abcam, ab654). Three random images of equal area containing GLUT4 immunostaining were selected from each slide, captured with a Leica DM500 light microscope and a Leica DFC290 HD camera (Leica Microsystems CMS GmbH, Wetzlar, Germany), and analyzed using ImageJ software. For each image, a threshold method was used to determine the number of brown pixels, corresponding to GLUT4 staining, relative to the total tissue area (interstitial spaces were excluded) and the ratio of GLUT4 area to muscle area was calculated and averaged for each animal. The researcher doing the pancreatic islet fibrosis ratio and GLUT4 quantification was blinded to the groups. Although a western blot would have quantitatively measured GLUT4 transporter in a more reproducible and sensitive manner that immunohistochemistry^43^, since muscle GLUT4 measurement was hypothesized after the rats’ sacrifice, no frozen muscle tissue was available to quantify GLUT4 using western blot.

### Statistical analysis

A univariate general linear model (ANCOVA) was used for data analysis. Final glucose AUC, GLUT4 ratio, pancreatic islet fibrosis ratio, plasma corticosterone and plasma insulin levels were included as dependent variables. The two nominal main factors established were infrasound exposure and glucose tolerance status. Duration of infrasound exposure and fasting insulin levels were included in this analysis as a covariate. The assumptions of normality variance homogeneity of the dependent variables were assessed using the Shapiro–Wilk test and the Levene test, respectively. Data analysis was performed with the software IBM SPSS Statistics for Windows, version 26 (IBM Corp., Armonk, NY, USA), at the 5% significance level (α = 0.05).

## Supplementary Information

Supplementary Information.
