# Supplementary material for: High-intensity infrasound effects on glucose metabolism in rats
Source: Sci Rep. 2021 Aug 26;11:17273. doi: 10.1038/s41598-021-96796-5 (PMC8390459; doi:10.1038/s41598-021-96796-5)
Supplement: Supplementary file 1 — Supplementary Information. [file 41598_2021_96796_MOESM1_ESM.docx]

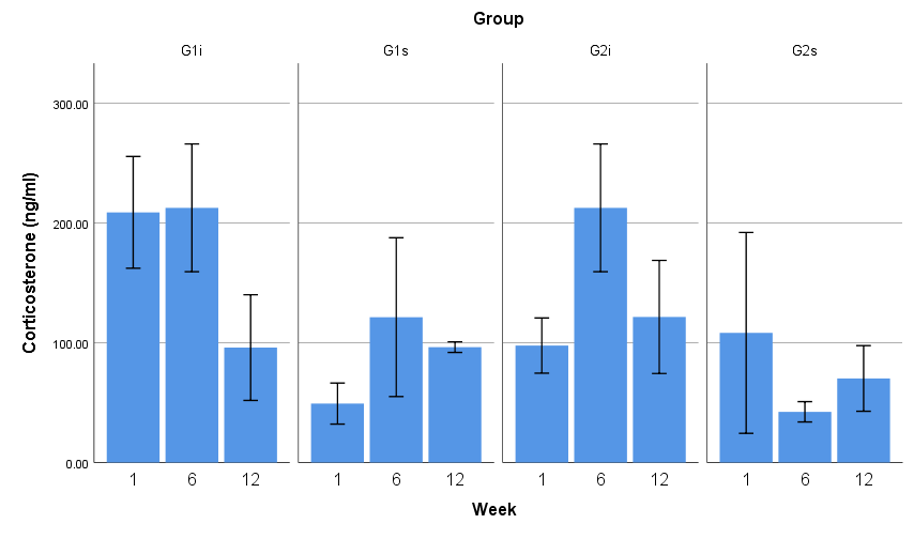


**Supplementary Material Figure 1 – Plasma corticosterone levels.** Means ± SE of plasma corticosterone levels (expressed as ng/ml), in normal (G1) and glucose intolerant (G2) animals, either kept in silence (s) or exposed to high-intensity infrasound (i), throughout the established experimental timepoints.


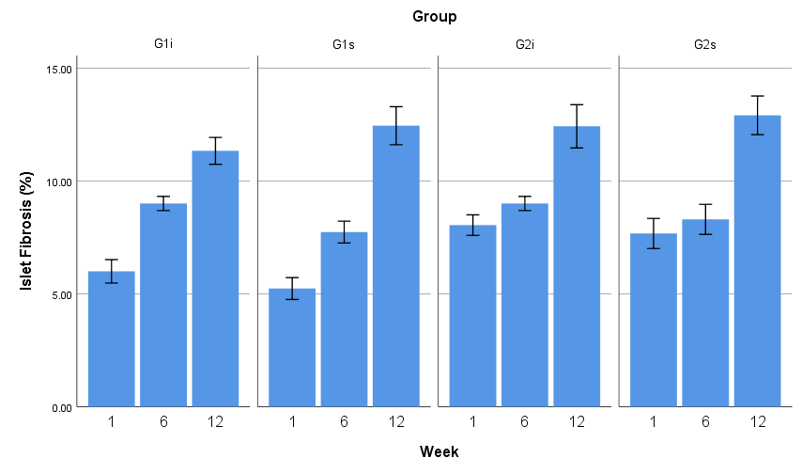


**Supplementary Material Figure 2 – Islet Fibrosis Ratio.** Means ± SE of islet fibrosis ratio (expressed as %), in normal (G1) and glucose intolerant (G2) animals, either kept in silence (s) or exposed to high-intensity infrasound (i), throughout the established experimental timepoints.
